# Climate Change Worry in Italian Young Adults: Psychosocial Predictors and Differences by Level of Environmental Activism Engagement

**DOI:** 10.11621/pir.2026.0101

**Published:** 2026-03-01

**Authors:** Daniela Acquadro Maran, Matteo Innocenti, Tatiana Begotti

**Affiliations:** a *University of Turin, Italy*; b Association for Climate Change Anxiety (AIACC), Italy

**Keywords:** climate worry, youth, environmental activism, self-efficacy, well-being, Italy

## Abstract

**Background:**

Climate change worry is an increasingly relevant emotional response among young adults. However, less is known about the psychosocial predictors of climate change worry and whether these associations differ by level of environmental activism engagement.

**Objective:**

This study examined psychosocial predictors of climate change worry in Italian young adults, focusing on mental health, personality traits, perceived individual and social norms, and individual and collective climate self-efficacy. A secondary aim compared more engaged versus less engaged participants.

**Method:**

Using convenience and snowball sampling via social media and word of mouth, 302 Italian young adults aged 18–35 years (*M* = 24.20, *SD* = 3.73; 62% female) completed an online survey. Group differences were tested using one-way ANOVAs. Multiple regression analyses were conducted separately in the more engaged (EA_MORE_) and less engaged (EA_LESS_) groups to identify predictors of climate change worry.

**Results:**

EA_MORE_ reported significantly higher climate change worry than EA-_LESS_. In EA_MORE_, climate change worry was predicted by mental health, collective self-efficacy, and perceived individual and social norms. In EA_LESS_, climate change worry was predicted by mental health and perceived individual and social norms, whereas collective self-efficacy was not significant.

**Conclusion:**

Climate change worry appears to reflect a combination of well-being and normative/efficacy-related processes, with different patterns depending upon the level of activism engagement. Supporting effective coping strategies may help reduce the emotional burden associated with sustained engagement.

## Introduction

Climate change and related environmental crises have proven to be not only environmental and socio-economic challenges, but also profound psychological stressors (Parmentier et al., 2024). As part of the growing number of psychological responses to environmental change, climate change worry (or eco-worry) has gained increased attention (Orrù et al., 2024). Climate change worry can be defined as a persistent form of concern or fear about environmental degradation and its current or future effects on ecosystems, human health and societal stability. As the literature analysis by Soutar and Wand (2022) shows, climate change worry is a component of ecoanxiety. The latter term was used by Clayton and Karazsia (2020) to describe a relatively intense form of anxiety, as opposed to a milder concern that can occur even without direct contact with the effects of climate change. However, as Pihkala (2020) suggests, it is important to avoid pathologizing experiences related to climate change perceptions. Anxiety can manifest along a spectrum of emotions that can range from mild to severe. The danger is to trivialize a term associated with a pathological manifestation that requires diagnosis and clinical intervention (Ojala et al., 2021; Bulbena-Vilarrasa & Bulbena-Cabré, 2024). In its severe form, anxiety may lead to panic attacks, obsessive thinking, appetite changes, and insomnia. These symptoms can significantly interfere with daily functioning (Léger-Goodes et al., 2022). Therefore, the term ‘eco-anxiety’ may not be the most appropriate to describe the negative feelings associated with climate change. Several scholars suggest that the term climate change worry is often more appropriate than eco-anxiety (see *e.g*. Ojala, 2012, 2019; Pihkala, 2020; Kurt & Akdur, 2024). Climate change worry can be considered an adaptive process, as it may motivate individuals to seek strategies for coping with environmental consequences (Innocenti et al, 2022; Cianconi et al., 2023). However, whether such concern is truly adaptive depends on its intensity and whether it triggers excessive negative emotions. Recent studies suggest that climate change worry is a multifaceted phenomenon. It may serve as a motivator for pro-environmental behavior, but it can also contribute to psychological stress, hopelessness, or maladaptive coping (Vecina et al., 2025). Understanding climate change worry is therefore crucial, both for mental health and as a potential driver for constructive engagement with environmental issues. Despite its relevance, empirical research on climate change worry remains relatively limited, and debates continue about its conceptual limitations, its measurement and its impact on well-being and behavior (López-García et al., 2025). Given the relevance of climate change worry for mental health and behavior, it is particularly important to examine how this phenomenon manifests among specific population groups.

As suggested by Ojala and Bengtsson (2019), young adults are the group most affected by the climate change narrative, probably because they will have to deal with it in the coming years and decades; they are also part of the climate change problem because of their lifestyle, as they are the consumers and citizens of today. There are personality traits that are vulnerability factors for climate worry, such as difficulty tolerating uncertainty (Taylor, 2020), negative emotionality (neuroticism; in particular in young people, see Ogunbode et al., 2024), tendency to overestimate threats, and sensitivity to fear (Tucholska et al., 2024).

Beyond socio-demographic exposure, previous research has suggested that individual differences may shape how climate change worry is experienced. Two basic individual characteristics (personal characteristics and mental health) and two individual dimensions specifically related to climate issues (individual and collective climate self-efficacy and perceived individual and social norms related to climate change) are considered. Mental health is considered in the present study as a general psychological condition that may influence individuals’ vulnerability to climate-related worry, rather than as an outcome of such worry. The theoretical framework into which the study fits is the Theory of Planned Behavior (TPB), a model developed by Ajzen (1991) that assumes that behavior is influenced by intention, which is shaped by attitudes, subjective norms and perceived behavioral control. In the present study, the TPB is used as a conceptual framework to justify the inclusion of norms and perceived behavioral control as psychosocial factors that may shape emotional responses to climate change, rather than behavior per se. In some studies (*e.g.* Fielding et al., 2008; Jew & Tran, 2022), the theory has been used to understand the intentionality of engagement in activism. More recently, Abonyi and McDermott (2024) conducted a study with 142 subjects in the UK and Hungary and highlighted that joining an environmental organization is associated with negative feelings and that belief in the ability to take action on climate change predicts all aspects of activism. Innocenti et al. (2023) suggested that individuals who perceive their own behavior, as well as others’ behavior, as effective in improving environmental health are more likely to adhere to pro-environmental norms. This adherence may translate into various forms of pro-environmental behavior, including activism. Bandura’s (1982) theory of self-efficacy can be summarized as the belief that one has the necessary resources and skills to perform or complete a particular task. The feeling of self-efficacy reflects people’s confidence that they can perform a behavior if they want to (Bain & Bongiorno, 2020). Self-efficacy has been shown to be a strong predictor of intention to act. In the case of environmentalists, self-efficacy is the belief that one has valuable skills to contribute to the achievement of group goals (Lenart-Gansiniec et al., 2022). When a group believes in its collective ability to learn, solve problems, or bring about change, its members are more likely to support one another (Vygostky, 1926, cited in Ratner, 1991). This process can foster engagement in collective environmental actions, such as participation in protest initiatives.

In addition to social and motivational factors, previous research has also examined the role of stable individual characteristics, such as personality traits, in shaping pro-environmental concern and behavior. Furthermore, the study conducted by Tucholska et al. (2024) with 333 participants in Poland showed that pro-environmental behavior is related to openness, a personality trait. Personality is defined as a characteristic and relatively stable pattern of thoughts, feelings and behaviors exhibited by individuals and provides an important explanation for individual differences in pro-environmental behavior. A meta-analysis based on 38 data sets of almost 50.000 people examined the relationships between the central personality dimensions of the Big Five model and showed the strongest correlations between openness, agreeableness, conscientiousness and - albeit to a lesser extent - extraversion with pro-environmental behavior. These results were later confirmed by Soutter and Mõttus (2021), who found the strongest correlations between openness and pro-environmental behavior, followed by agreeableness and conscientiousness. At the same time, extraversion and neuroticism showed low correlations with pro-environmental behavior.

Overall, openness proves to be the most influential personality trait in the expression of pro-environmental behavior. This trait is associated with abstract and flexible thinking, which enables individuals to grasp the complex temporal and spatial consequences of the climate crisis. In addition, openness can indirectly promote activism by influencing the values that individuals hold.

In this study, climate change worry is conceptualized as a psychological outcome whose level may vary as a function of individual, social, and motivational factors. Accordingly, the study adopts a predictor-oriented approach, focusing on the psychosocial factors associated with different levels of climate change worry. The main objective of this study was to compare climate change worry in two groups of young adults living in Italy, based on their engagement in environmental activism. In Italy, a survey from 2019 found that more than half of a sample of 800 young adults cited climate change as the main cause of stress (SWG, 2019), while the ISTAT report (2025) shows that 116.628 young adults are engaged in environmental volunteerism. The two groups were compared with respect to several personal variables. These included personality traits and mental health, as well as climate-related dimensions such as individual and collective self-efficacy and perceived individual and social norms. The second aim of the study was to analyze the relationship between the above-mentioned personal variables and climate change worry, separately for participants that are “more” (EA_MORE_) or “less” (EA_LESS_) engaged in environmental activism.

Based on the above-mentioned literature, we hypothesised that EA_MORE_ perceive higher climate change worry than EA_LESS_ (*Hp. 1*) and that they are characterized by openness, agreeableness and conscientiousness (*Hp. 2a*). At the same time, we hypothesised that EA_LESS_ — more than EA_MORE_ — are characterized by extraversion and neuroticism (*Hp. 2b*). We also hypothesised that EA_MORE_ perceive a better mental health status (*Hp. 3*), a higher level of individual and collective self-efficacy (*Hp. 4*) and a higher level of individual and social norms (*Hp. 5*) than EA_LESS_. *Hypothesis 3* was formulated as theoretically exploratory, as previous research has provided mixed findings on the association between environmental activism and mental health, and no clear directional expectation has been consistently established.

## Methods

Based on this conceptual framework, climate change worry was examined as an outcome variable, while personality traits, mental health, perceived norms, and climate self-efficacy were included as psychosocial predictors.

### Participants

A total of 308 young adults living in Italy participated in the study by completing an anonymous online questionnaire. Of the participants, 30% identified as male, 62% as female, and 8% preferred not to report their gender. Participants’ ages ranged from 18 to 35 years (M = 24.20, SD = 3.73). Participation was voluntary, and no compensation was provided.

### Measures

Participants completed an anonymous online questionnaire. The instrument was specifically developed for the purposes of the study and administered using the LimeSurvey platform. Convenience and snowball sampling strategies were used.

Environmental Activism was measured through 7 items assessing the engagement of the participants in different activities during the last year. The items were taken from the Environmental Action Scale (EAAS; Alisat & Riemer, 2015). As suggested by Clayton and Parnes (2025), the selected items refer to specific areas: participation in or organization of educational activities; contact with elected officials; raising awareness of climate change on public platforms (*e.g.*, letters to the editor, social media); participation in or organization of protests, rallies, boycotts, and/or petitions; financial support for an environmental or climate-related cause; and engagement (*e.g.*, volunteering, working) or building local coalitions (*e.g.*, “In the past year ... I have volunteered with an environmental organization working on climate issues”). For the purposes of our study, the following answers were possible for each question: yes (*coded as 2*) or no (*coded as 1*). The items were totaled and the mean was calculated (M = 9.67; SD = 2.18). Participants who scored above the mean value were considered EA_MORE_ (meaning that they answered “yes” to at least three of the items).

Climate change worry was measured through a scale consisting of 10 items adapted from the work of Stewart (2021) and the Italian version edited by Innocenti et al. (2022). Participants were asked to indicate the degree of worry about several issues, such as: perceiving to experience a worry about climate change more than other people; having a tendency to worry when hearing about climate change, even when the effects might be far away; believing that bad weather might be a consequence of climate change; difficulty in their ability to stop thinking about climate change. For each question, possible responses were: strongly disagree (*coded as 1*), disagree (*coded as 2*), neither agree nor disagree (*coded as 3*), agree (*coded as 4*), and strongly agree (*coded as 5*). Range: 10–50. Cronbach’s alpha = .88.

Personality Traits were measured through the Brief Big Five Personality Inventory-10 (BFI-10)- (John, Donahue & Kentle, 1991). It consisted of a 10-item scale (developed based on the 44-item Big Five Inventory) measuring the Big Five personality traits: Extraversion, Agreeableness, Conscientiousness, Emotional Stability, and Openness (2 items for each personality trait). For each item possible responses were: strongly disagree (*coded as 1*), in disagreement (*coded as 2*), neither agree nor disagree (*coded as 3*), in agreement (*coded as 4*), strongly agree (*coded as 5*). Range of each personality trait: 2–10.

Mental health was assessed through the WHO-5 Mental Health Index: this scale is an abbreviated version of the WHO-10, developed by the World Health Organization (Bech et al., 1996). It consists of 5 items inherent to subjective mental well-being. The WHO-5 has only positive valence expressions, as well-being is considered an expression of good mental health. Participants had to indicate the answer that most closely resembled how they have been feeling for the past two weeks (*for example*, cheerful and in a good mood; calm and relaxed; active and energetic). Possible responses were: never (*coded as 0*), sometimes (*coded as 1*), less than half-time (*coded as 2*), more than half-time (*coded as 3*), most of the time (*coded as 4*), and always (*coded as 5*). Range: 0–25. Cronbach’s alpha = .79.

Climate self-efficacy (Individual and Collective) was assessed through the Perceived Climate Self-Efficacy Scale (Doran, Hanss & Larsen, 2015; 2017). Participants were asked to indicate the extent to which they agreed with a series of statements, 5 of which related to individual self-efficacy (*for example,* ability to motivate others to do something for the climate crisis; ability to make an important contribution to climate change mitigation through personal actions) and 5 of which related to collective efficacy (*for example*, belief that young people can contribute to solving the climate crisis; belief that young people can help protect the climate through efforts to influence climate legislation). For each question, possible responses were: strongly disagree (*coded as 1*), disagree (*coded as 2*), neither agree nor disagree (*coded as 3*), agree (*coded as 4*), and strongly agree (*coded as 5*). Range of each scale: 5–25. Cronbach’s alpha = .78 and .81 respectively.

Perceived individual norms related to climate change were measured using 2items (Doran & Larsen, 2016) aimed to assess participants’ perceived concern for the environment as an important part of themselves and their existence as a young person. Possible responses were: strongly disagree (*coded as 1*), disagree (*coded as 2*), neither agree nor disagree (*coded as 3*), agree (*coded as 4*), and strongly agree (*coded as 5*). Range: 2–10. Cronbach’s alpha = .76.

Perceived social norms related to climate change were measured using 8 items related to worry and fears about climate change and its future impacts (Doran & Larsen, 2016). Participants were asked the extent to which the statements applied to people they care about (*4 items*) and to people of the same age (*4 items*). Possible responses were: strongly disagree (coded as 1), disagree (*coded as 2*), neither agree nor disagree (*coded as 3*), agree (*coded as 4*), and strongly agree (*coded as 5*). Range: 8–40. Cronbach’s alpha = .84.

### Procedure and data analysis

The local ethical committee of the University of Turin approved this research project (protocol code 0421423). The sample was recruited with the help of platforms such as LinkedIn, Instagram* (specifically contacting *Fridays For Future* pages) and through word of mouth. The data were collected between July and September 2023. The participants completed an anonymous online questionnaire accompanied by an information letter and an informed consent form. The compilation took about 20 minutes.

Data were processed with SPSS version 28 (IBM Corp., Armonk, NY, USA). Reliability of the measures was assessed with Cronbach’s alpha. One-way ANOVA analyses were used to test group differences between EA_MORE_ and EA_LESS_ participants, whereas multiple regression analyses were conducted to examine the psychosocial predictors of climate change worry. Eta squared was calculated to estimate effect size.

## Results

### Descriptive statistics

Participants were classified as more or less engaged in environmental activism based on their position relative to the sample mean. This criterion was adopted as an operational and distribution-based approach to distinguish higher versus lower levels of engagement, rather than to define discrete activist categories. Accordingly, this grouping should be interpreted as reflecting relative differences in engagement within the sample. Based on the seven items measuring Environmental Activism we divided the sample into two groups (excluding 6 subjects with missing data): EA_MORE_ (those who reported being involved in at least three different environmental activism activities; N = 127; 42%) and EA_LESS_ (those who reported being involved in less than three different environmental activism activities; N = 175; 58%). The use of a mean-based cut-offallowed the inclusion of participants with intermediate levels of engagement in the analytical comparison, while avoiding overly restrictive categorizations.

The level of climate change worry was compared between the two groups using a one-way ANOVA analysis. To test *Hypothesis 1*, a one-way ANOVA was conducted to compare levels of climate change worry between young adults who were more engaged (EA_MORE_) and less engaged (EA_LESS_) in environmental activism. For results, see *Table 1*.

**Table 1:** Climate change worry: comparison between EA_MORE_ and EA_LESS_

	**EA_MORE_ (N=127) M (SD)**	**EA_LESS_ (N=175) M (SD)**	**F**	**P**	**η^2^**
Climate change worry	39.00 (6.07)	34.02 (7.33)	38.03	<.001	.114

*Note. M = mean; SD = standard deviation; F = Fisher’s ratio; p = p value; η^2^ = eta squared.*

**Table 2:** Personality traits and mental health: comparison between EA_MORE_ and EA_LESS_

	**EA_MORE_ (N=127) M (SD)**	**EA_LESS_ (N=175) M (SD)**	**F**	**p**	**η^2^**
Extraversion	6.36 (1.74)	6.22 (1.75)	.513	.475	.002
Agreeableness	6.58 (1.58)	6.27 (1.61)	2.612	.107	.009
Conscientiousness	6.62 (1.50)	6.77 (1.28)	.875	.350	.003
Neuroticism	6.56 (1.85)	6.46 (2.03)	.158	.691	.001
Openness	6.92 (1.59)	7.03 (1.45)	.394	.531	.001
Mental Health	14.74 (4.22)	16.39 (4.06)	11.67	<.001	.038

*Note. M = mean; SD = standard deviation; F = Fisher’s ratio; p = p value; η^2^ = eta squared.*

The results support *Hypothesis 1*, indicating significantly higher levels of climate change worry among EA_MORE_.

To test *Hypotheses 2a, 2b*, and *3*, a one-way ANOVA was performed to compare EA_MORE_ and EA_LESS_ with respect to personality traits and mental health. For results, see *Table 2*.

As we can see from the results shown in *Table 2*, there are no significant differences between EA_MORE_ and EA_LESS_ with respect to the five personality traits. In contrast, a significantly higher level of mental health is found in EA_LESS_. *Hypotheses 2a* and *2b* were not supported, as no significant differences emerged for personality traits. *Hypothesis 3* was not supported, as EA_LESS_ participants reported significantly higher levels of mental health.

Next, variables more specifically related to the issue of climate were examined.

To test *Hypotheses 4* and *5*, a one-way ANOVA was performed to compare EA-_MORE_ and EA_LESS_ on individual and collective climate self-efficacy and perceived individual and social norms related to climate change. For results, see *Table 3*.

**Table 3:** Individual and collective climate self-efficacy, perceived individual and social norms related to climate change: comparison between EA_MORE_ and EA_LESS_

	**EA_MORE_ (N=127) M (SD)**	**EA_LESS_ (N=175) M (SD)**	**F**	**p**	**η^2^**
Individual self-efficacy	18.35 (3.25)	16.57 (3.75)	17.47	<.001	.057
Collective self-efficacy	19.65 (2.82)	18.09 (3.64)	15.36	<.001	.050
Individual norms	8.22 (1.53)	6.94 (1.62)	46.01	<.001	.136
Social norms	27.61 (4.86)	26.77 (5.28)	1.94	.165	.007

*Note. M = mean; SD = standard deviation; F = Fisher’s ratio; p = p value; η^2^ = eta squared.*

As we can see in *Table 3*, EA_MORE_ show significant higher levels than EA_LESS_ in both individual and collective climate self-efficacy and in perceived individual norms related to climate change. No differences were found concerning social norms. *Hypothesis 4* was supported, whereas *Hypothesis 5* was only partially supported.

To find out how climate change worry was related to personality traits, mental health, individual and collective climate self-efficacy, perceived individual and social norms related to climate change in EA_MORE_ and EA_LESS_, correlation analyses were performed separately in the two groups. *Table 4* reports the results for EA_MORE_ and EA_LESS_ respectively.

As reported in *Table 4,* concerning personality traits, we can see that climate change worry is negatively related to agreeableness, and positively related to neuroticism and openness only in EA_MORE_. Moreover, climate change worry is positively related to individual and collective self-efficacy, individual and social norms both in EA_MORE_ and EA_LESS_.

**Table 4:** Correlations analysis in EA_MORE_ and EA_LESS_

	**Climate change worry EA_MORE_**	**Climate change worry EA_LESS_**
Extraversion	–.09	.03
Agreeableness	–.22*	.01
Conscientiousness	–.036	.09
Neuroticism	.22*	.08
Openness	.29**	–.007
Mental Health	.28**	.20**
Individual self-efficacy	.30**	.33**
Collective self-efficacy	.28**	.40**
Individual norms	.50**	.41**
Social norms	.28**	.25**

To further examine the psychosocial predictors of climate change worry, multiple regression analyses were conducted separately for EA_MORE_ and EA_LESS_. Multiple regression analyses were performed in EA_MORE_ and EA_LESS_ separately, to assess the possible effects on climate change worry of the variables found to be significant in the correlation analysis. *Tables 5* and *6*show the results for EA_MORE_ and EA_LESS_ respectively. The regression model explained approximately 40% of the variance for EA_MORE_ (R^2^ = .42) and 30% for EA_LESS_ (R^2^ = .29).

**Table 5:** Regression analysis in EA_MORE_. Dependent variable: climate change worry

	**β**	**T**	**P**
Agreeableness	–.06	–.67	.51
Neuroticism	.07	.87	.39
Openness	.01	.08	.94
Mental health	.16	2.04	.04
Individual self-efficacy	.09	–.82	.41
Collective self-efficacy	.25	2.45	.02
Individual norms	.52	6.60	.001
Social norms	.15	2.01	.047

*Note. R^2^ = 42.*

As shown in *Tables 5* and *6*, in the case of EA_MORE_, mental health, collective self-efficacy, perceived individual and social norms (the latter at the limit of statistical significance) were found to be predictors of climate change worry, whereas in the case of EA_LESS_, mental health and perceived individual and social norms were significant predictors.

**Table 6:** Regression analysis in EA_LESS_. Dependent variable: climate change worry

	**β**	**T**	**p**
Mental health	.19	2.81	.006
Individual self-efficacy	.14	1.36	.18
Collective self-efficacy	.03	.27	.79
Individual norms	.31	4.26	.001
Social norms	.21	3.08	.002

*Note. R^2^ = .29*

## Discussion

The present study analysed the differences between young Italian adults who are more engaged in environmental activism (EA_MORE_) and their peers who are less engaged in environmental activism (EA_LESS_) in order to understand the predictors of climate change worry in a group of young adults in Italy. The present findings should be interpreted in light of the theoretical framework outlined in the Introduction, which conceptualizes climate change worry as a multifaceted psychological outcome shaped by individual, social, and motivational factors. The results show that EA_MORE_ report significantly higher levels of worry about climate change than EA_LESS_, which confirms the *Hp.1* and is consistent with the literature linking active engagement with increased risk awareness and urgency regarding climate change (Khatibi et al., 2021).

Regarding personality traits (*Hp.2a* and *Hp.2b*), the data showed no significant differences between the two groups, which refutes the hypotheses formulated on the basis of the meta-analyses by Soutter & Mõttus (2021), which pointed to positive associations between openness, kindness and conscientiousness and pro-environmental behavior. The absence of significant differences in personality traits suggests that dispositional characteristics may play a limited role in distinguishing levels of environmental engagement when compared to social and motivational factors, such as norms and self-efficacy. Furthermore, the results suggest that participation in pro-environmental behavior is determined more by motivational and situational variables (*e.g*. norms, self-efficacy) than by stable dispositional differences. The *Hp.3*, which predicted better mental health in EA_MORE_, was not confirmed: EA_LESS_ reported significantly higher mental well-being. These findings run counter to initial expectations. While environmental activism is associated with motivation and self-efficacy, it may also involve psychological costs linked to prolonged exposure to climate-related stressors (Maran & Begotti, 2021; Léger-Goodes et al., 2022; Pihkala, 2020). *Hypothesis Hp.4*, which refers to the perception of higher level of individual and collective self-efficacy among EA_MORE_, was confirmed. Rather than functioning as a buffer against emotional distress, higher collective self-efficacy among more engaged participants may reflect increased perceived responsibility and emotional involvement in addressing the climate crisis. In this sense, self-efficacy may amplify climate change worry by reinforcing personal and collective accountability. EA_MORE_ reported greater confidence in their own abilities and those of the group, which is consistent with Bandura’s (1982) theory and recent studies identifying self-efficacy as a crucial predictor of participation in environmental activism (Abonyi & McDermott, 2024; Innocenti et al, 2023). Finally, Hp.5 was only partially confirmed: EA_MORE_ reported higher levels of individual norms, but no significant differences from EA_LESS_ in social norms. This finding may reflect the increasing prevalence of climate change worry in the adolescent population, regardless of the level of activist engagement (Clayton & Parnes, 2025). The results thus suggest that while internalized norms differentiate the choice of activism, climate-related social norms appear to be an overarching feature. Regression analyses were used to further elucidate the predictors of climate change worry in the two groups. In EA_MORE_, climate change worry is explained by mental health, collective self-efficacy, individual norms and (at most) social norms, confirming the role of collective and normative dimensions in supporting activation. In EA_LESS_, however, the significant predictors are mental health and (individual and social) norms, suggesting that the experience of climate change worry is more related to general perceptions of well-being and normative belonging than to processes of collective efficacy. This fact can be explained by the perception that not everyone shares concern for the well-being of the environment. For example, research by Timmons et al. (2024) shows that many young people already underestimate how worried older generations are about climate change and that this misperception is linked to the belief that others will not come together to tackle climate change.

Overall, the results show that climate worry among young adults is an ambivalent phenomenon. On the one hand, it can serve as a driver for activism and strengthen processes of self-efficacy and normative internalization; on the other hand, it can have a negative impact on psychological well-being, especially for those who become more engaged with the climate issue through activism.

## Conclusion

Building on the existing literature on climate-related emotions and environmental engagement, this study contributes to clarifying the psychosocial factors associated with climate change worry among young adults. The results show that young adults more engaged in environmental activism report greater worry about climate change, accompanied by higher levels of individual and collective self-efficacy and stronger individual norms. This confirms the idea that worry about climate change can have an adaptive and motivational function that promotes pro-environmental action. However, the value for mental health reverses expectations: EA_MORE_ report lower scores than EA_LESS_, suggesting that continued preoccupation with the climate crisis also has psychological costs. Furthermore, the two groups do not differ in terms of social norms, suggesting that worry for the climate is now widespread among young people, regardless of level of engagement in environmental activism. Overall, the hypotheses were only partially confirmed: while self-efficacy emerges as a central factor, personality traits do not appear to make a difference between activists and non-activists, and mental health appears to be more at risk among the most highly engaged. These findings highlight the importance of considering climate change worry not only as a potential risk to well-being, but also as a possible driver of environmental engagement. Supporting effective coping strategies may help reduce the emotional costs associated with activism.

To effectively address the impact of climate change on mental health and wellbeing, Lawrance et al. (2022) argue that it is essential that individuals and communities are adequately equipped and supported. This includes strategies aimed at facilitating psychological and emotional processing of experiences such as grief, loss, and fear, while promoting hopeful perspectives. It is also important to support the ability of people and social groups to imagine and envision a desirable future in order to prevent the exacerbation of existing psychological distress. Building a sense of community, developing functional adaptation strategies and promoting individual and collective agency are key elements in strengthening psychosocial resilience. Finally, active engagement in tackling the climate crisis can contribute significantly to strengthening social cohesion and a sense of shared efficacy.

## Limitations

The present study has some methodological and interpretative limitations. Firstly, the sample is not representative of the Italian youth population: data collection through social networks and platforms linked to environmental movements may have favored the participation of participants who are already sensitized to the issue. Secondly, due to the cross-sectional nature of the research design, it is not possible to establish causal relationships between the variables, which limits the possibility of understanding whether climate change worries precede or result from activism. To better understand the process linking the variables, future research could utilize a longitudinal design that measures whether climate change worry increases or decreases as a result of respondents’ engagement before and during environmental activism. Thirdly, our study includes young adults who are engaged in environmental activism to varying degrees. In doing so, we did not examine some variables (*e.g.,* political beliefs, motivation, and individual values) that might be useful for understanding the reasons for engagement in these activities. Future research could benefit from examining the principles that inspire engagement in environmental activism. Another limitation concerns the use of self-reporting, which can be influenced by social desirability, particularly in relation to pro-environmental behaviors and active engagement. Future research could incorporate social desirability instruments (on this topic, see Oerke & Bogner, 2013). Moreover, particularly among young people, to whom media coverage of this topic is dedicated, climate change worry can be perceived as an issue that is considered essential, making it impossible not to declare oneself worried in some way. This is also evident from the work of Benoit et al. (2022) on media coverage of climate change. Newspaper articles often portray children and young people and their reflective perspectives and experiences of climate change as young activists and saviors of the planet. Thus, the analysis did not consider relevant contextual variables such as the extent of media exposure and perceived social support which could influence the intensity of climate change worry (see Maran & Begotti, 2021). Future studies could therefore also take media exposure and social media presence into account. In addition, the use of the BFI-10, while useful for brevity, reduces reliability in the multifaceted measurement of personality traits. In future research, the adoption of more comprehensive psychological well-being measures may provide a more nuanced understanding of the relationship between climate change worry and psychological functioning. Specifically, multidimensional well-being scales could capture positive psychological functioning more precisely than general mental health indices, thereby enriching the interpretation of emotional responses to climate change. More broadly, future research could also incorporate alternative personality models that increase measurement accuracy; for example, Feher and Vernon (2021) suggest the use of the HEXACO model when examining morally relevant outcomes.

## Footnotes

* Activities of Meta Platforms Inc. (Facebook and Instagram), and the X Corp. (as the successor of Twitter Inc.), are prohibited in the Russian Federation.
